# Early Discharged Lumbar Spine Fusion Reduced Postoperative Readmissions: A Retrospective Cohort Study

**DOI:** 10.3390/ijerph17041335

**Published:** 2020-02-19

**Authors:** Wen-Cheng Huang, Jau-Ching Wu, Hsuan-Kan Chang, Yu-Chun Chen

**Affiliations:** 1 Department of Neurosurgery, Neurological Institute, Taipei Veterans General Hospital, Taipei 11217, Taiwan; wchuang@vghtpe.gov.tw (W.-C.H.); jauching@gmail.com (J.-C.W.); hsuankanchang@gmail.com (H.-K.C.); 2 School of Medicine, National Yang-Ming University, Taipei 11221, Taiwan; 3 Department of Biomedical Imaging and Radiological Sciences, National Yang-Ming University, Taipei 11221, Taiwan; 4 Department of Family Medicine, School of Medicine, National Yang-Ming University, Taipei 11221, Taiwan; 5 Department of Family Medicine, Taipei Veterans General Hospital, Taipei 11217, Taiwan; 6 Institute of Hospital and Health Care Administration, National Yang-Ming University, Taipei 11221, Taiwan

**Keywords:** lumbar spinal fusion, early discharge (ED), incidence, readmission, reoperation, risk management

## Abstract

Early discharge (ED) has emerged and gained popularity in spine surgery. However, the benefits of ED in lumbar fusion have not yet been validated by large cohort studies. To evaluate the effects of ED on readmissions and reoperations in lumbar fusion, this study utilized a national database to enroll patients who had undergone lumbar fusion surgery at age 50–70 years, and grouped them into an ED group or a comparison group. In the comprehensive follow-up of 180 days post-operation, the two groups were compared. There were 18,008 patients in the cohort, including 2172 in the ED group and 15,836 in the comparison group. The ED group was slightly younger (59.9 vs. 60.7 years, *p* < 0.001), more male predominant (44.9% vs. 36.9%, *p* < 0.001), and had fewer medical comorbidities. The ED group had less incidences of readmission than the comparison group. (Crude hazard ratio = 0.73, and adjusted HR = 0.75, both *p* < 0.001). Overall, the cumulative incidences of readmission in the ED group (9.5%) were lower than those in the comparison group (12.8%, *p* < 0.001), whereas reoperations were insignificantly different (1.5% vs. 1.2%, *p* = 0.189). For patients aged 50–70 years and who require lumbar fusion surgery, ED could yield a 25% reduced risk of readmission for any cause within 180 days post-operation. Since the reoperation rates remained similar, our results suggest that ED may be a promising option for elderly patients undergoing lumbar spinal fusion surgery.

## 1. Introduction

Lumbar spinal arthrodesis is one of the most common fusion surgeries of the spine performed in the modern era. There have been many emerging technologies, such as minimally invasive approaches, low profile instrumentations, and biologics, to facilitate the process of recovery during the past couple of decades [1,2,3,4,5,6]. The concept of early discharge (ED) has also been introduced into the field of spine surgery in the past several years [7,8,9,10,11]. By incorporation of strategic management peri-operatively, the early discharge (ED) approach has aimed to raise the efficiency of the entire treatment process so that the patients could recover better [12,13,14]. The attempts at using this patient-centered ED approach to improve lumbar fusion procedures have demonstrated promising preliminary results in recent years [7,11,15]. However, more data are required to corroborate the adaptation of ED in spinal surgery.

The current study aims to investigate differences between patients who were discharged early and those who were not following lumbar fusion surgery, focusing on the rates of readmission and complications that required secondary surgery. Since the rates of success and patient satisfaction for common lumbar fusion surgery have become high in the past decades [16,17], the need for readmission or reoperation was low in each surgeon’s or institute’s series [18,19,20,21,22]. Therefore, there was rarely an adequate number of patients with high enough rates of follow-up for investigation of readmissions or reoperations in lumbar spinal fusion surgery. The current study, using a large cohort, takes advantage not only of a large number of patients, but also mitigates many of the confounders by statistical adjustment. The readmissions and reoperations could reflect the most clinically relevant outcomes of lumbar spinal fusion surgery. 

Using the National Health Insurance Research Database (NHIRD) of Taiwan, the study features extremely high follow-up rates, comprehensive coverage of all health care providers, and unrestricted access to medical services of patients enrolled [23,24]. Therefore, the study uniquely enables investigation of the outcomes of lumbar fusion surgery in a national insurance system. To date, it is the largest cohort study focusing on the effects of ED on incidences of readmission and reoperations after lumbar spinal fusion surgery. 

## 2. Material and Methods

### 2.1. Data Source and Ethical Concerns

This population-based retrospective cohort study used admission records of Taiwan’s National Health Insurance Research Database (NHIRD). The NHIRD comprehensively contains de-identified claim data for Taiwan’s National Health Insurance (NHI) program, which covers 99% of the Taiwanese population and contracts with 97% of the providers of healthcare services in Taiwan. In order to protect privacy, the National Health Research Institute (NHRI) re-compiled, validated, and de-identified the medical claims and finally made the data publicly available for medical researchers in Taiwan. In the admission database, we were able to trace comprehensive information on the insured subjects, including gender, date of birth, dates of clinical visits and hospitalization, the International Classification of Diseases (Ninth Revision) Clinical Modification (ICD-9-CM) codes of diagnoses, ICD codes of surgical procedures, etc. 

This study protocol was approved by the Institutional Review Board of Taipei Veterans General Hospital (IRB# 2018-09-0006CC). The Institutional Review Board waived the requirement for written informed consent from each of the patients involved since all identifying personal information in the NHIRD was encrypted. The study protocol was registered in Clinical Trails.gov (ClinicalTrials.gov ID: NCT04126980).

### 2.2. Study Cohort

**Figure 1:**
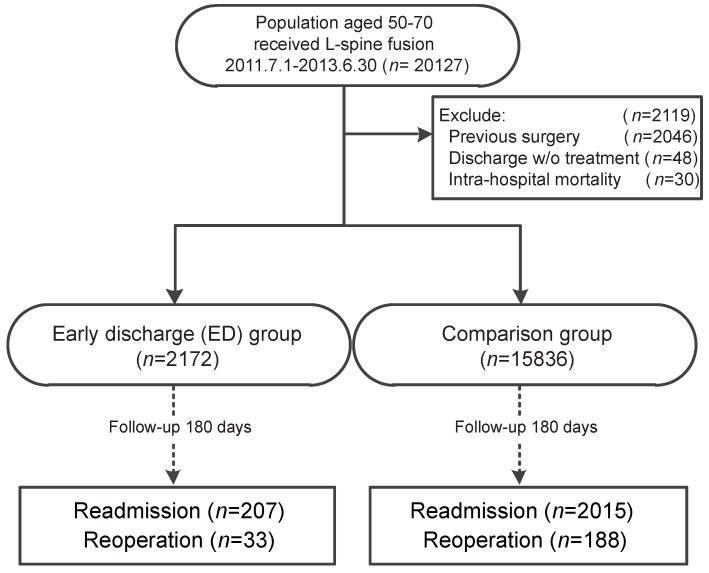
Flowchart of data processing in a cohort of patients who received first lumbar spinal fusion surgery. Patients were grouped into two: the early discharge (ED) group and the comparison group. Initial patients may have met one or more exclusion criteria; therefore, the total number exceeded the number of included patients (*n* = 18,008).

We enrolled patients who had received lumbar spine fusion (ICD-9 operative procedure: 81.05, 81.08, 81.35, 81.38) between 1 July 2011 and 30 June 2013. To minimize confounders of age and previous spinal disorders, only patients aged between 50 and 70 years old without any spinal problem, such as any spinal fusion (ICD-9 operative procedure: 81.0, 81.3, and 81.6) or spinal disc operations (ICD-9 operative procedure: 84.51, 84.52, 84.59, 84.6, and 84.8), were included for subsequent analysis. This strict inclusion could better quantify the effect of ED, due to the homogeneity of the study group. However, the study design might limit the implication of results for other age groups of patients or more complicated surgical approaches (Figure 1). 

For each admission in our study cohort, we designated the first day of hospitalization as the index date for the lumbar spine fusion, and the length of admission for hospitalization was calculated. We further limited our study cohort to a length of admission of between one and twelve days to reduce outliers (e.g., complicated surgery) and to make our study cohort more homogenous and comparable. Patients who had received any prior spinal surgery, or who were discharged from hospitalization without surgery, or who experienced intra-hospital mortality were excluded from the analysis.

### 2.3. ED Group vs. Comparison Group

All patients were assigned to either the ED group or the comparison group according to the length of stay for hospitalization. Patients in eastern Asian countries typically have a longer length of stay for any surgery requiring hospitalization due to cultural reasons, welfare issues, and the payment system. They are often admitted 1 or 2 days prior to the surgery for pre-operative evaluations and anesthesiology preparation. Therefore, the median length of stay for a routine electively performed lumbar spine fusion would be approximately 11 days, including 1 full day pre-operative preparation prior to the surgery. However, since 2010, Taiwan’s Bureau of National Health Insurance (BNHI) has gradually implemented the Diagnosis-Related Group (DRG) payment system for 1062 major surgeries, including spinal fusion, to give medical facilities greater incentive to reduce medical resource expenditures (BNHI, 2013). Since then, spine surgeons have started to adopt the strategy of ED to increase the efficiency of health care service and reduce the length of admission for lumbar spinal fusion. 

To compare the outcome of ED to conventional management strategies, we assigned patients who were hospitalized, successfully operated on, and discharged in less than 72 h as the ED group, while patients who had a length of hospitalization longer than 72 h but less than 12 days were assigned to the comparison group. Subsequently, both the ED and comparison groups were analyzed.

### 2.4. Follow-Up Outcomes and Covariates

The most clinically related outcomes of lumbar spinal fusion, a common neurosurgical procedure with extremely high patient satisfaction, were readmissions and reoperations. These two outcome parameters were also likely to be associated with neurological functions post-operation. Since the NHIRD uniquely provided a very comprehensive follow-up of the entire cohort, all patients in the study were followed-up for 180 days. Any readmission and reoperation within 180 days after the indexed surgery would be tracked, even if they were in different institutes, or physically distant, owing to the universal coverage of the government-supported monopolistic health insurance scheme. Because all the medical care providers were contracted with the BNHI and allowed all patients unrestricted access, any post-operative events would very likely be captured by the NHIRD. Due to the rigorously monitored billing processes, the readmissions and reoperations of these patients would have little chance of being lost to follow-up. 

All-causes of re-hospitalization and reoperations for lumbar spine fusion were analyzed at 30, 60 and 180 days for comparison between the ED group and the comparison group. To accurately evaluate the effects of ED, we included the 10 most prevalent comorbidities as controlling variables. These medical comorbidities included anemia, chronic peptic ulcer disease, chronic pulmonary diseases, depression, diabetes, hypertension, chronic hepatic diseases, congestive heart failure, neurologic disorders, and chronic kidney disease. 

### 2.5. Statistical Analysis

All the data were linked using the SQL server 2017 (Microsoft Corp, Redmond, WA, USA) and analyzed by Stata software (Stata Corp, College Station, TX, USA). The Kaplan-Meier method and a log-rank test were used to estimate and compare cumulative readmission and reoperation rates among different groups. Adjusted hazard ratio (aHR) for readmission and reoperation for each factor was estimated by controlling other factors in the model. A two-tailed level of 0.05 was considered statistically significant. 

## 3. Results

A total of 18,008 patients who underwent lumbar spinal fusion surgery between July 2011 and June 2013 were identified in and extracted from the NHIRD (Figure 1). Among them, 2172 patients were categorized into the ED group, who had less than 72 h of hospitalization (including the preparation period which commonly took approximately one day) for the surgery. The other 15,836 patients were categorized as the comparison group, who were hospitalized for more than 72 h but less than 12 days. All patients were then followed-up for 180 days after the indexed surgery of lumbar fusion. 

### 3.1. Demographics of the Cohort

The comparison group and the ED group had some differences in gender composition, age, and medical comorbidities (Table 1). Among the 18,008 patients analyzed, the ED group (*n* = 2172) were slightly younger (59.9 vs. 60.7 years, *p* < 0.001) and more male predominant (44.9% vs. 36.9%, *p* < 0.001) than the comparison group (*n* = 15,836). 

The ED group had fewer medical comorbidities, including anemia, chronic pulmonary diseases, congestive heart failure, diabetes, hypertension, and liver diseases than the comparison group. In the group of ED patients, there was less anemia (3.9% vs. 6.6%, *p* < 0.001), less chronic pulmonary disease (12.4% vs. 15.6%, *p* < 0.001), less congestive heart failure (2.2% vs. 3.4%, *p* = 0.003), less diabetes (24.0% vs. 27.8%, *p* < 0.001), less hypertension (40.8% vs. 45.0%, *p* < 0.001), and less liver disease (12.8% vs. 14.6%, *p* = 0.032), when compared to that of patients in the comparison group. There were no statistical differences between the ED and comparison groups in depression (4.7% vs. 5.7%, *p* = 0.057), neurologic disorders (2.0% vs. 2.7%, *p* = 0.084), chronic kidney diseases (1.4% vs. 1.7%, *p* = 0.340), and chronic peptic ulcer disease (4.9% vs. 5.2%, *p* = 0.620). 

### 3.2. Readmission Rates

All-cause readmissions were less in the ED group than the comparison at 30 days, 60 days, and 180 days post-operation. However, reoperations on the lumbar spine were not significantly different between the ED and comparison groups at 30 days, 60 days, and 180 days post-operation (Table 1). 

After adjustment was made (for age, gender, and all the comorbidities), all-cause readmission was significantly less in the ED group (Table 2). Patients of the ED group were 0.73 or 0.75 times less likely to need readmission for any cause. (Crude hazard ratio = 0.73, whereas adjusted HR = 0.75, both *p* < 0.001). The incidences of reoperation did not reach significance for the ED group when compared to that of the comparison group. (Crude HR = 1.28, whereas adjusted HR = 1.28, both *p* > 0.05). Thus, ED reduced by 25–27% the chance of readmission compared to the comparison group. (Crude HR = 0.73, and adjusted HR = 0.75, both *p* < 0.001).

**Figure 2:**
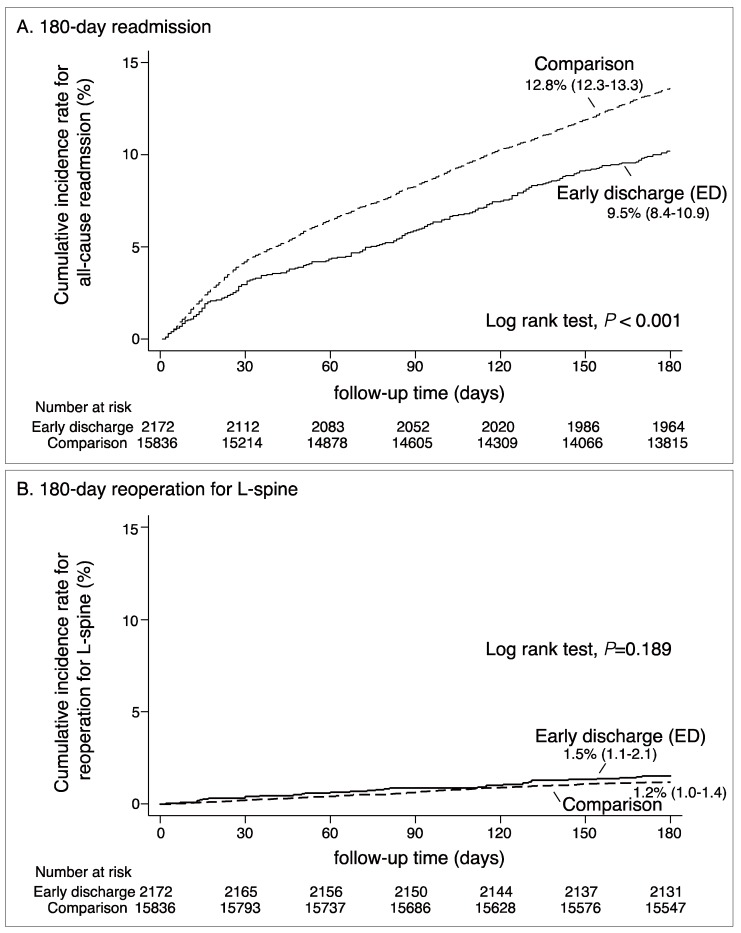
Cumulative incidence rate for 180-day all-cause readmission and 180-day reoperation after lumbar spinal fusion surgery by patient groups. Patients were grouped into two: the early discharge (ED) group and the comparison group (*n* = 18,008).

Generally, one out of ten patients (about 10%) had been hospitalized after their index lumbar spinal surgeries and a portion of patients (1%) even had secondary surgery on their spine. The cumulative incidences of readmission in the ED group (9.5%) were lower than that in the comparison group (12.8%, *p* < 0.001), whereas reoperations were insignificantly different in both groups (1.5% vs. 1.2%, *p* = 0.189) (Figure 2).

## 4. Discussion

This cohort study used the NHIRD of Taiwan, a unique database that covers the entire country, to analyze the incidence of readmission and reoperation after lumbar fusion surgery. The patients who underwent strategical management to achieve ED for the lumbar fusion surgery were compared to those who could or did not. There were 18,008 patients in the cohort, including 2172 in the ED group and 15,836 in the comparison group. The analysis demonstrated that the ED group was less likely (cHR = 0.73, and aHR = 0.75) to need readmission than the comparison group, while the reoperation rates were not significantly different between the two groups. Therefore, for lumbar fusion surgery, ED should be advocated, since it reduced the rate of all-cause readmissions by at least 25%. This was the first study to demonstrate the huge advantage of ED for patients aged 50–70 years who underwent lumbar fusion procedures.

The study results indicated that ED could lower the rate of readmission. Although the study design incorporated strategies to minimize confounders, further studies are warranted for validation of the causal effect rather than sheer coincidence. The current study design deliberately adopted a large cohort of middle-aged (50–70 years) patients, with the exclusion of previous spinal surgery and other disorders to reduce selection bias. However, there were still chances of bias from both the surgeons and patients. For instance, the surgeons might recruit healthier patients for ED and patients with more comorbidities as the control group. Also, the patients who agreed to be discharged earlier in the postoperative course of their disease might simply have less concerns for their health than others and may therefore have an overall lower readmission rate. It is reasonable to infer that ED is an effective management strategy in certain subgroups, while for others it may not be [25]. Therefore, even though the current study demonstrated benefits of ED, more investigation focusing on different subgroups and pooling more observational studies from other sites are necessary to corroborate this.

The strategical management of ED for lumbar spine surgery involves a combination of technologies to enhance recovery, accelerate discharge, and ultimately reduce medical expenses by designated intervention in pre-, intra-, and post-operations [11]. Moreover, an enhanced version of ED program-concerted institutional-level efforts to reduce the burden of surgery through a combination of changes in practice, called Enhanced Recovery After Surgery (ERAS), was proposed [26]. For example, ERAS-transforaminal lumbar interbody fusion (TLIF) advocated by Wang et al. adopted multimodal technology to maximize patient recovery in a very short period following lumbar fusion surgery, a procedure notorious for post-operative pain and the need for hospitalization [15,27]. Their technique combined an exceedingly minimally-invasive procedure and instruments, including intravenous anesthesia without intubation, endoscopic discectomy, expandable mesh cages, percutaneous pedicle screws, and a regional long-acting analgesic agent [7,28,29]. Their results demonstrated early discharge and durable outcomes for patients undergoing ERAS-TLIF surgery, and successfully addressed the principle of the ED program for lumbar spine. Preliminary studies have encouraged the possibility of enhanced recovery in lumbar spine operations. Although the report demonstrated the superior outcomes of adopting ED for lumbar fusion surgery, the technology is not universal. The success of ED is dependent on selecting appropriate patients for the advanced surgical techniques of lumbar fusion. That remains the key in patient management.

There have been arguments over whether the length of hospital stay (LOS) truly reflects patients’ recovery. Functional outcomes have been suggested to be a more appropriate assessment. To date, very few studies have evaluated patients’ functional recovery as a primary outcome (as opposed to LOS) as it is difficult to justify [30]. LOS remains a fundamental outcome measurement in evaluating the ED program. Future ED studies in spine surgery should focus on function status as an outcome assessment. Although short-term success has been demonstrated in the ED program for lumbar spine surgery, longer-term results have never been investigated before. Our study analyzed a national-scale database and concluded that patients’ early discharge achieved results as good as conventional management in terms of readmission and reoperation. Patients’ outcomes and safety would not be jeopardized by early discharge after lumbar spine surgery. Moreover, although the length of hospitalization usually depends on the patients’ recovery time, it could also be affected by personality of the patients or their families, labor power of care, or medical condition post-operation.

The study was limited by several issues. There were still some heterogeneities that could not be controlled, such as indications of the lumbar fusion surgery, levels of lumbar fusions achieved, surgical techniques applied, and neurological outcomes. Modern lumbar fusion surgery involves a great variety of surgical approaches, including posterior transforaminal fusion, lateral interbody fusion, endoscopic approaches, and minimally invasive surgery. Although none of these were specifically accounted for in the outcomes in the current study, the authors asserted tremendous efforts to adjust the other comorbidities. All the other medical comorbidities that might affect the outcomes were adjusted statistically. In other words, the 25% gain (aHR = 0.75) demonstrated in the ED patients was independent of older age, cardiac problems, or any other medical comorbidities. Therefore, this study suggests that elderly patients who need lumbar fusion surgery may benefit from ED management. Furthermore, in the current study, the ED group was identified by an ultra-short length of stay and lacked a clear definition of its management protocol. In fact, this cohort study involved hundreds of hospitals and medical centers, and therefore it was impossible for all the spine surgeons involved to utilize a uniform ED program. Nevertheless, the ED group of patients demonstrated remarkable advantage in readmission rates. This warrants future investigations to look for the optimal ED protocol for each kind of lumbar fusion surgery.

## 5. Conclusions

For patients aged 50 to 70 years who require lumbar fusion surgery, ED could yield a 25% reduced risk of readmission for any cause within 180 days post-operation. Since reoperation rates remained similar, ED should be advocated for such patients of lumbar spinal fusion surgery.

## Figures and Tables

**Table 1:** Demographic characteristics, comorbidities, and outcomes of comparison and early discharge (ED) groups, 2011–2013 (*n* = 18,008).

Demographic Characteristics, Comorbidities, and Outcomes				Comparison Group		ED ^1^ Group		*p*-Value
				*n* = 15,836	(%)	*n* = 2172	(%)	
	Gender							<0.001
		Female		9988	(63.1)	1196	(55.1)	
		Male		5848	(36.9)	976	(44.9)	
	Age, mean (SD ^2^)			60.7	(5.74)	59.9	(5.76)	<0.001
	**Comorbidities**							
		Anemia		1041	(6.6)	85	(3.9)	<0.001
		Chronic pulmonary disease		2463	(15.6)	269	(12.4)	<0.001
		Congestive heart failure		535	(3.4)	47	(2.2)	0.003
		Depression		910	(5.7)	103	(4.7)	0.057
		Diabetes		4406	(27.8)	522	(24.0)	<0.001
		Hypertension		7120	(45.0)	887	(40.8)	<0.001
		Liver disease		2307	(14.6)	279	(12.8)	0.032
		Neurologic disorders		420	(2.7)	44	(2.0)	0.084
		Chronic kidney disease		270	(1.7)	31	(1.4)	0.340
		Chronic peptic ulcer disease		820	(5.2)	107	(4.9)	0.620
	**Outcome**							
		All-cause readmission						
			30-day readmission	636	(4.0)	64	(2.9)	0.016
			60-day readmission	962	(6.1)	89	(4.1)	<0.001
			180-day readmission	2015	(12.7)	207	(9.5)	<0.001
		Re-operation for L-spine						
			30-day re-operation	33	(0.2)	9	(0.4)	0.062
			60-day re-operation	66	(0.4)	14	(0.6)	0.130
			180-day re-operation	188	(1.2)	33	(1.5)	0.190

^1^ ED: early discharge. ^2^ SD: standard deviation.

**Table 2:** All-cause readmission rates and reoperation rates for L-spine during 180 day follow-up of comparison and early discharge (ED) groups after index L-spine fusion, 2011–2013. (*n* = 18,008).

Readmission during 180-Day-Follow-Up			Comparison Group	ED Group		
**All-cause readmission**						
		Incidence of readmission (per 1000 person-years)	275.2	201.7		
		Number of occurrences	2015	207		
		Observed person-years	7320.9	1026.3		
		Crude hazard ratio (95% C.I.) ^1^	1.00	0.73	(0.63–0.85) ***^,3^	
		Adjusted hazard ratio (95% C.I.) ^2^	1.00	0.75	(0.65–0.87) ***^,3^	
**Reoperation for L-spine**						
		Incidence of reoperation (per 1000 person-years)	24.0	30.7		
		Number of occurrences	188	33		
		Observed person-years	7842.1	1075.1		
		Crude hazard ratio (95% C.I.) ^1^	1.00	1.28	(0.86–1.86)	
		Adjusted hazard ratio (95% C.I.) ^2^	1.00	1.26	(0.87–1.83)	

^1^ C.I., confidence interval. ^2^ Adjusted for age, gender, anemia, chronic pulmonary disease, congestive heart failure, depression, diabetes, hypertension, liver disease, neurologic disorders, chronic kidney disease, and chronic peptic ulcer disease. ^3^ Significance level: ***, *p* < 0.001.
